# Supplementary material for: In Vitro Evaluation of the Antifungal Activity of Trigonella foenum-graecum Seed Extract and Its Potential Application in Plant Protection
Source: Plants (Basel). 2025 Oct 31;14(21):3320. doi: 10.3390/plants14213320 (PMC12608566; doi:10.3390/plants14213320)
Supplement: Supplementary file 1 [file plants-14-03320-s001.zip › plants-3949641-supplementary.pdf]

Supplementary material

*Submitted to* Special Issue

# *In vitro* evaluation of the antifungal activity of *Trigonella foenum-graecum* seed extract and its potential application in plant protection

**Stelica Cristea[1], Alina Perisoara[2*], Bianca-Maria Tihauan[3,], Manuela Diana Ene[2], Mariana Constantin[4,5], Alexandru-Mihai Florea[2], Elena Ştefania Ivan[1], Relu Cristinel Zală[1], Bogdan Purcareanu[2,6], Dan Eduard Mihaescu[7] and Lucia Pirvu[8]**

[1] University of Agronomical Sciences and Veterinary Medicine, 59 Mărăşti Blvd., District 1, 011464, Bucharest, Romania, stelicacristea@yahoo.com (S.C), elena.ivan@qlab.usamv.ro (E.I.), cristinel.zala@usamv.ro (R.C.Z)
[2] Biotehnos SA., 3-5 Gorunului Street, 075100 Otopeni, Ilfov County, Romania, pavel.alinaa@gmail.com (A.P.), alexandru.florea@biotehnos.com (A.M.F) , bogdanpb89@gmail.com (B.P), diana.ene@biotehnos.com (M.D.E)
[3] Research Institute of the University of Bucharest-ICUB, Splaiul Independenței, no. 95, District 5, Bucharest, Romania, ciubuca.b@gmail.com (B-M.T.)
[4] National Institute for Research & Development in Chemistry and Petrochemistry-ICECHIM, 202 Independentei Spl., 060021 Bucharest, Romania, marriconstantin@yahoo.com
[5] Faculty of Pharmacy, Titu Maiorescu University, 16 Bd. Gh. Șincai, 040441 Bucharest, Romania
[6] Department of Science and Engineering of Oxide Materials and Nanomaterials, National University of Science and Technology Politehnica Bucharest, 011061 Bucharest, Romania, bogdanpb89@gmail.com
[7] Department of Organic Chemistry, National University of Science and Technology Politehnica Bucharest, 011061 Bucharest, Romania, danedmih@gmail.com
[8] National Institute for Chemical-Pharmaceutical Research and Development, District 3, Bucharest, Romania, lucia.pirvu@yahoo.com
* Correspondence: pave.alinaa@gmail.com

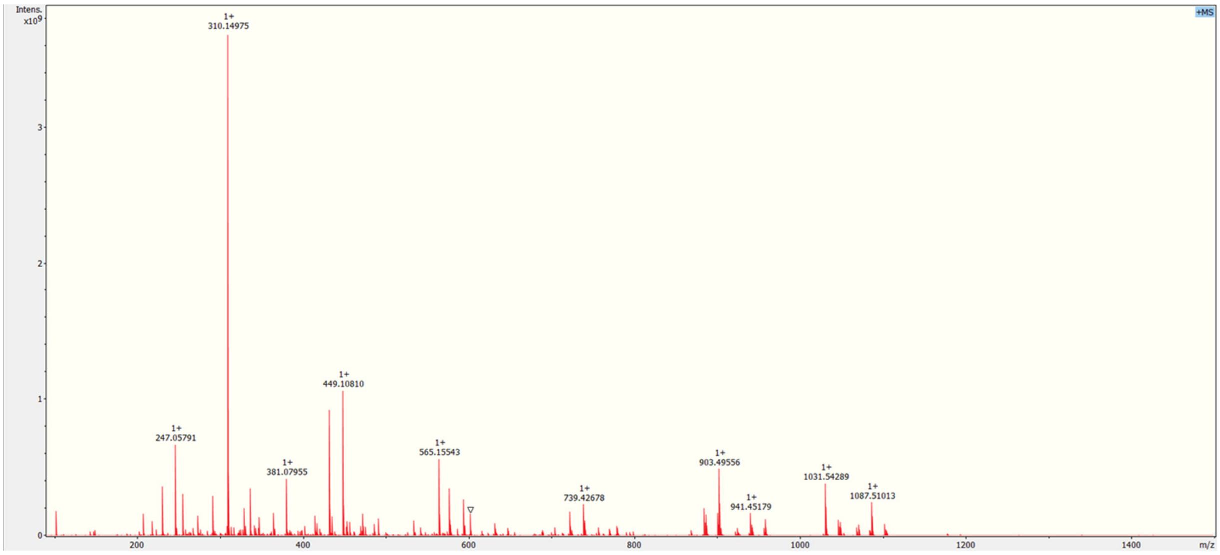


**Figure S1.** Full mass spectra of fenugreek extract using positive ESI ionization

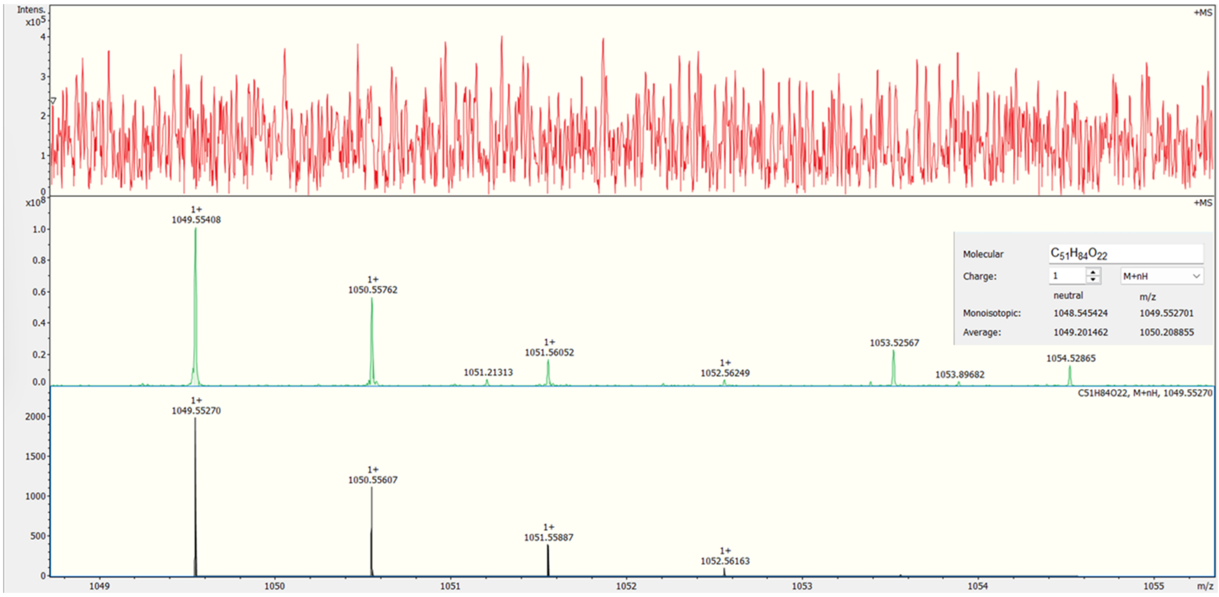


**Figure S2.** Molecular peak for Protodiosgenina ($C_{51}H_{84}O_{22}$) – m/z calculated is 1049.552, ESI positive

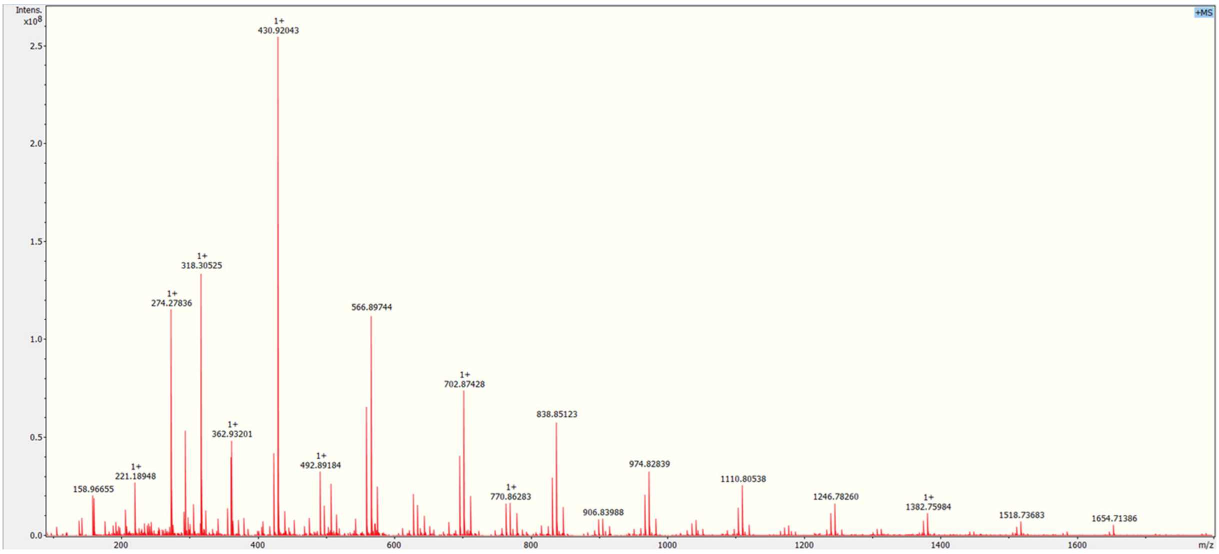


**Figure S3.** Full mass spectra of Sodium trifluoroacetate using positive ESI ionization for the calibration of the instrument

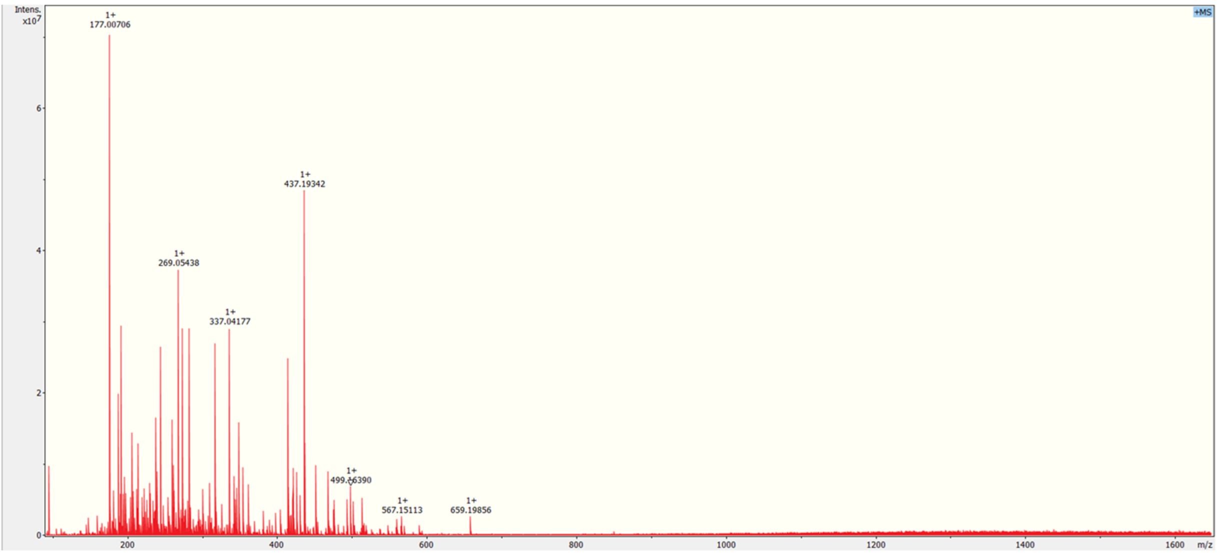


**Figure S4.** Full mass spectra of blank sample using positive ESI ionization

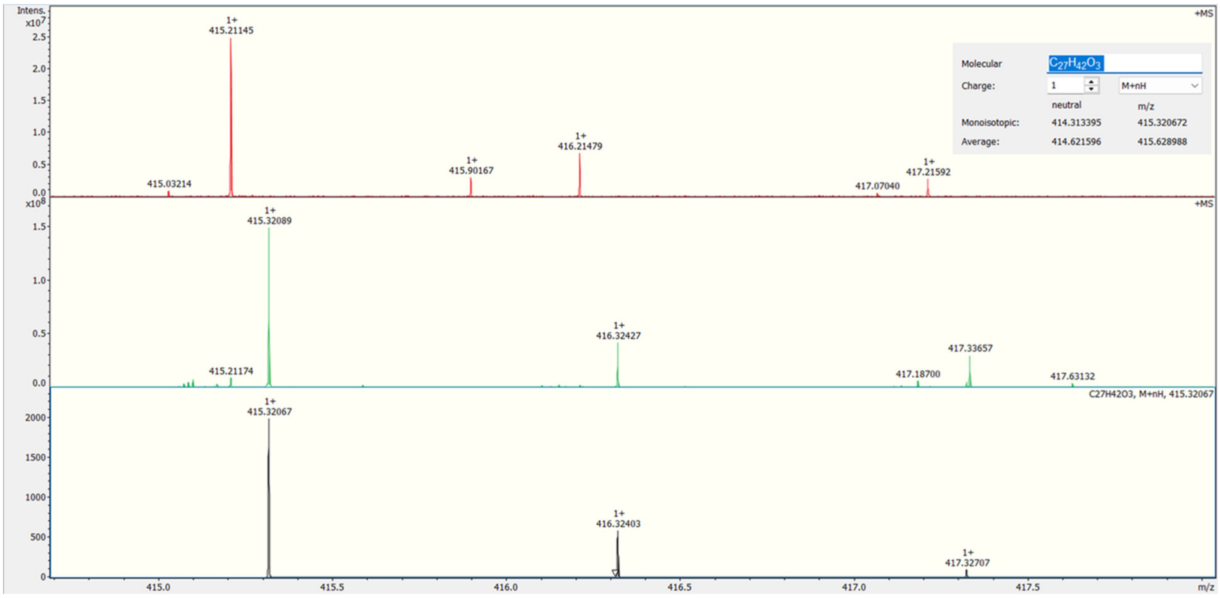


**Figure S5.** Molecular peak for Diosgenina ($C_{27}H_{42}O_3$) – m/z calculated is 415.320, ESI positive

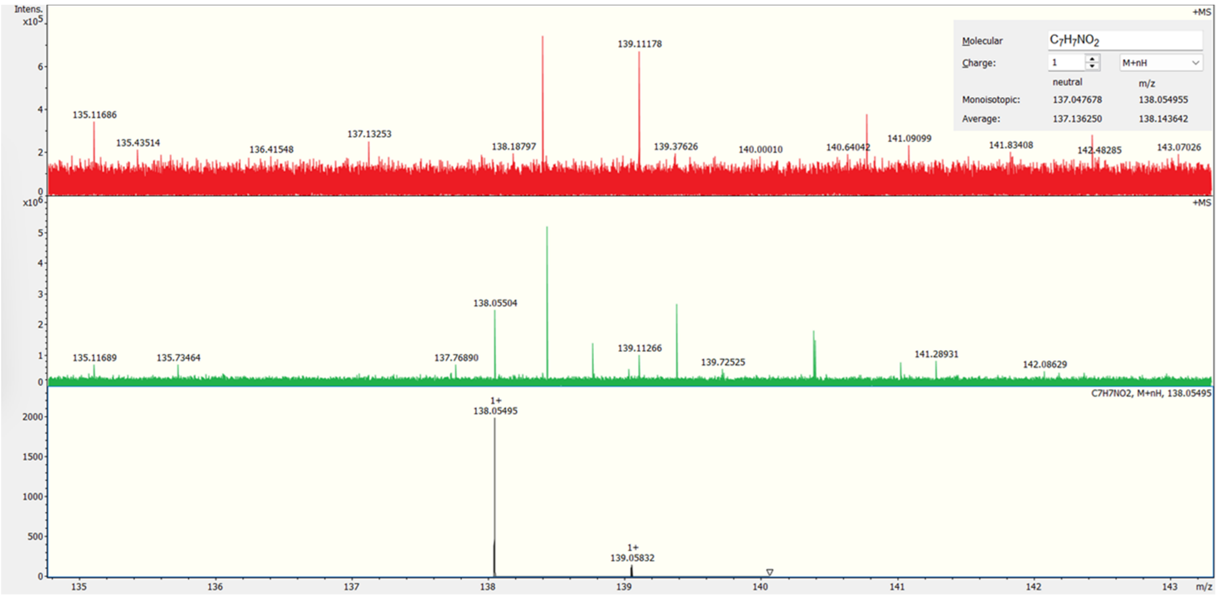


**Figure S6.** Molecular peak for Trigonelline ($C_7H_7O_2$) – m/z calculated is 138.054, ESI positive

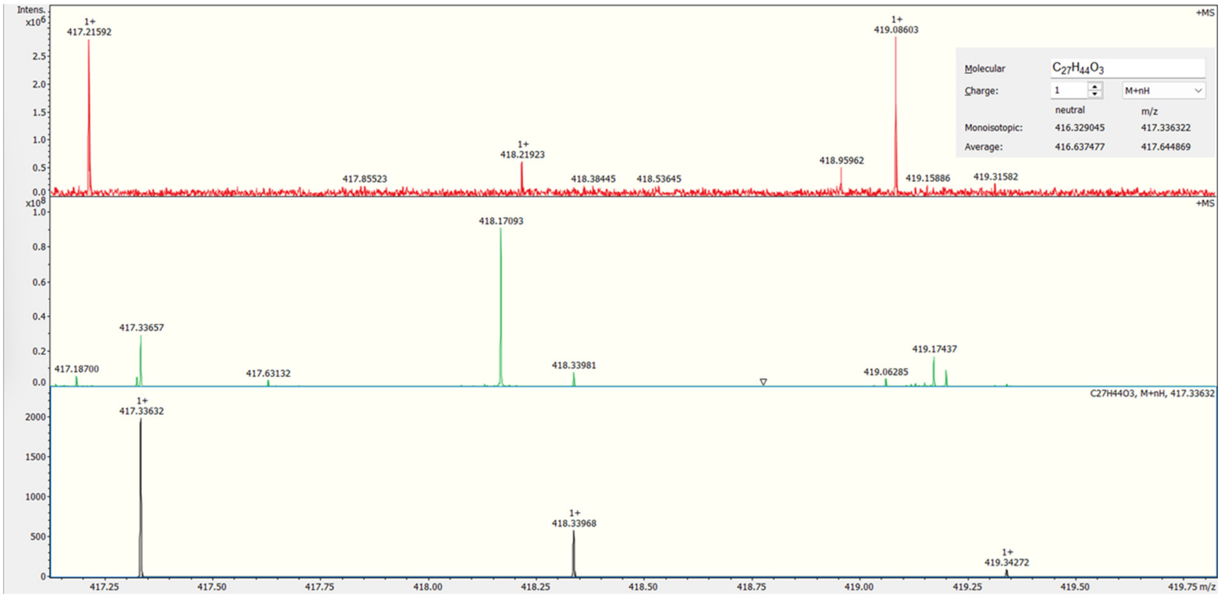


**Figure S7.** Molecular peak for Tigogenina ($C_{27}H_{44}O_3$) – m/z calculated is 417.336, ESI positive

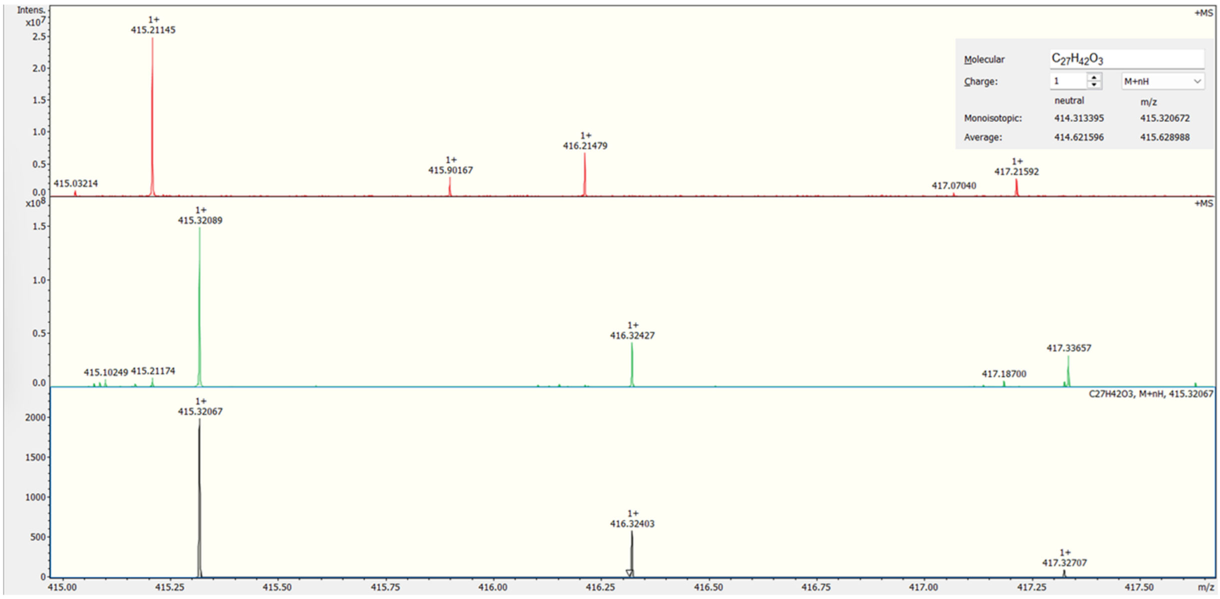


**Figure S8.** Molecular peak for Yamogenina ($C_{27}H_{42}O_3$) – m/z calculated is 415.320, ESI positive

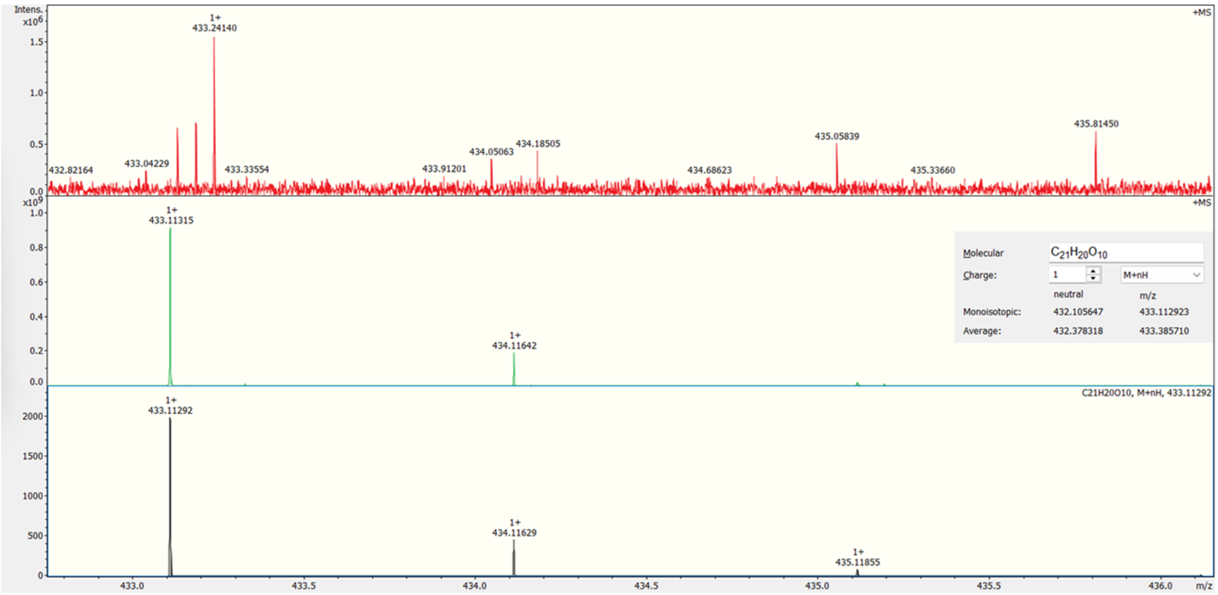

**Figure S9.** Molecular peak for Vitexin ($C_{21}H_{20}O_{10}$) – m/z calculated is 433.112, ESI positive

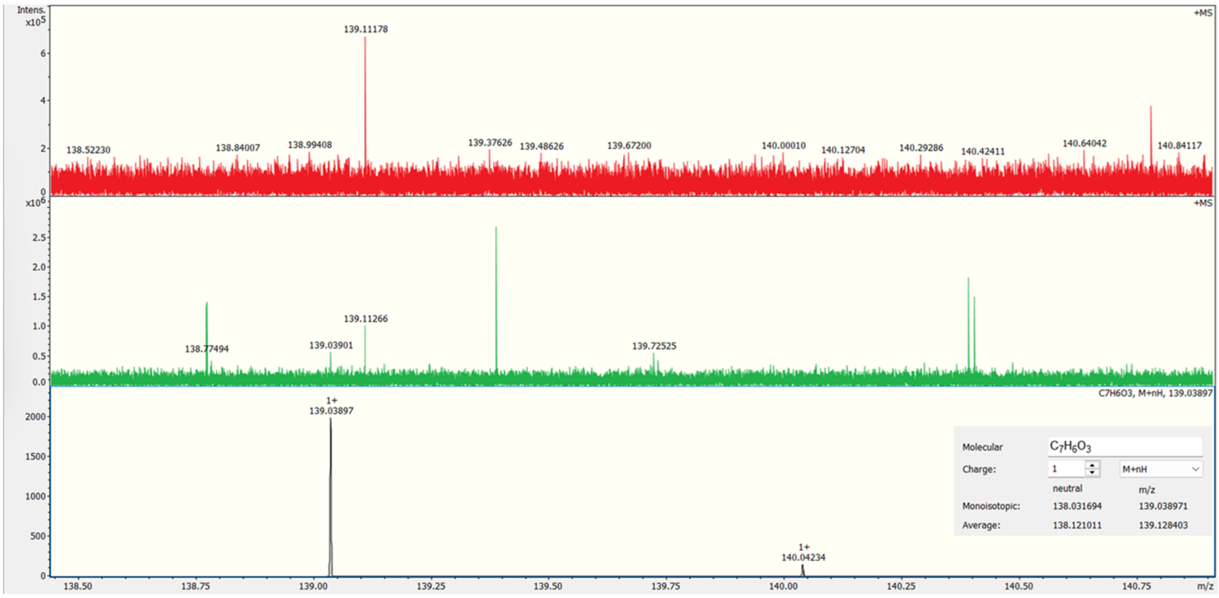

**Figure S10.** Molecular peak for Acid salicylic ($C_7H_6O_3$) – m/z calculated is 139.038, ESI positive

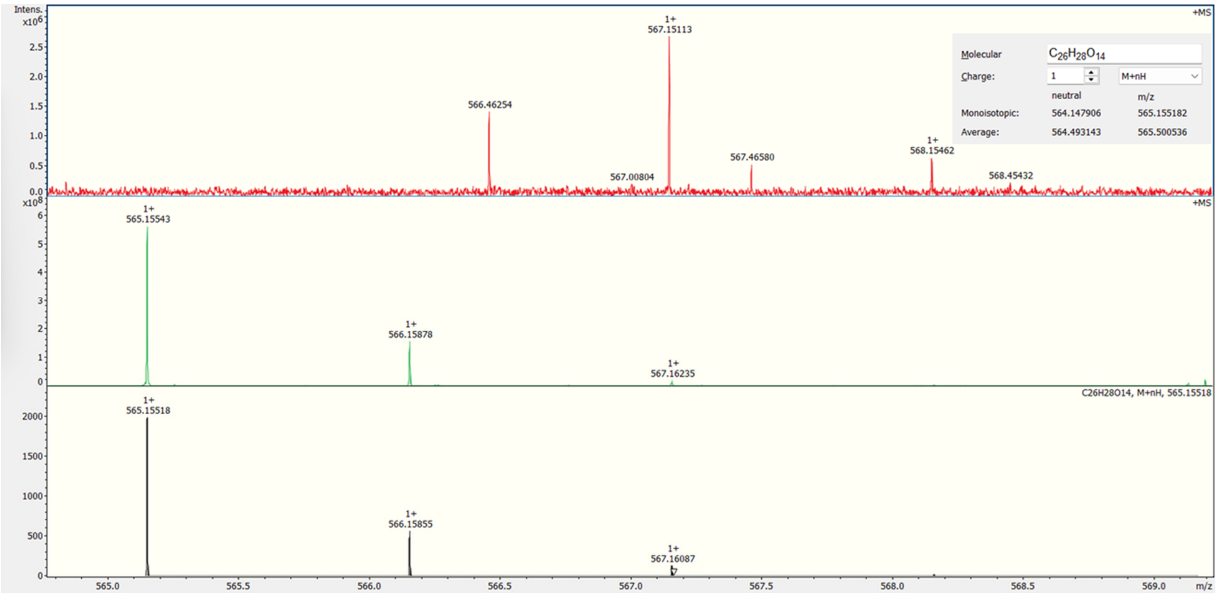


**Figure S11.** Molecular peak for Apigenin 6-C-galactoside 8-C – arabinoside ($C_{26}H_{28}O_{14}$) – m/z calculated is 565.115, ESI positive

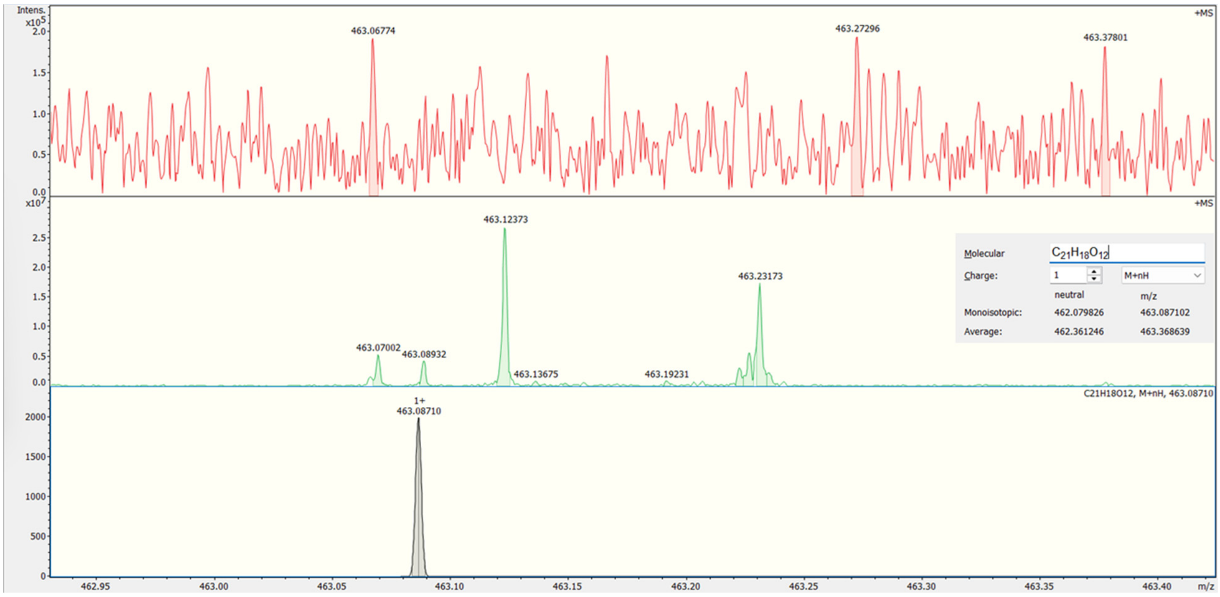


**Figure S12.** Molecular peak for Luteoline – 7 – O glucoside ($C_{21}H_{18}O_{12}$) – m/z calculated is 463.087, ESI positive

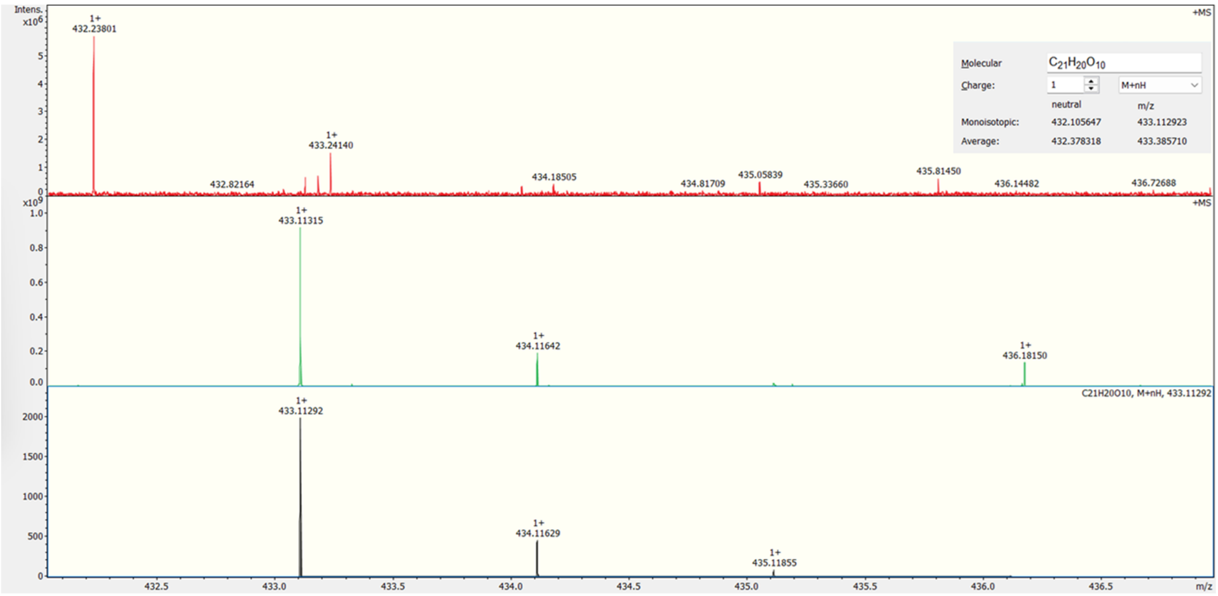


**Figure S13.** Molecular peak for Apigenin – 7 – O – glucoside ($C_{21}H_{20}O_{10}$) – m/z calculated is 433.112, ESI positive

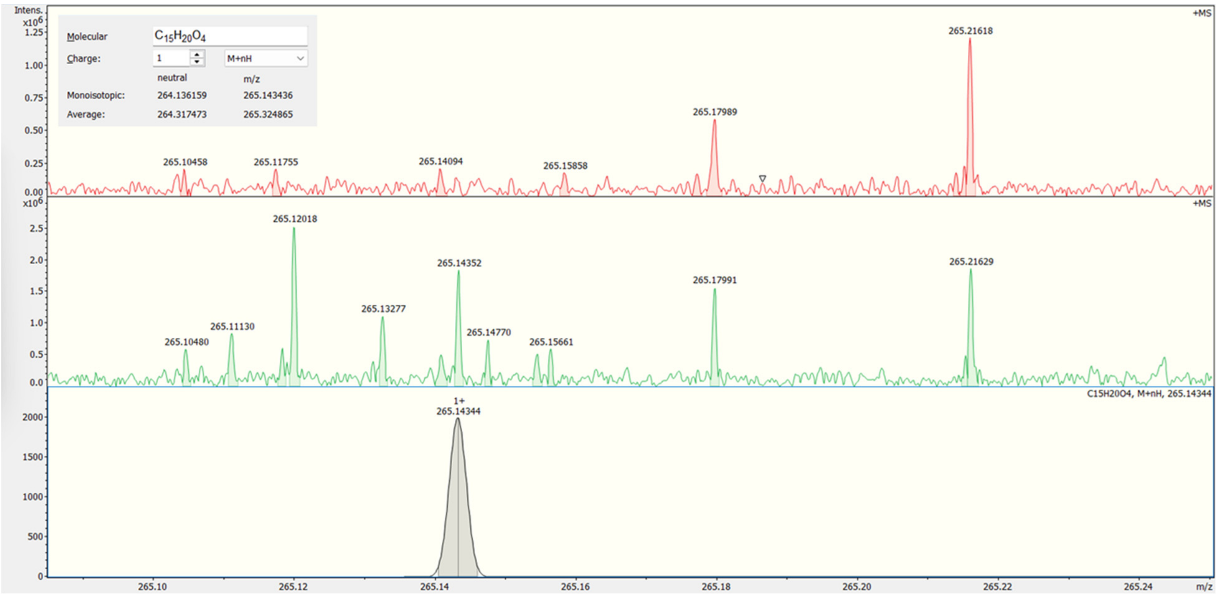


**Figure S14.** Molecular peak for Acid abscisic ($C_{15}H_{20}O_4$) – m/z calculated is 265.143, ESI positive

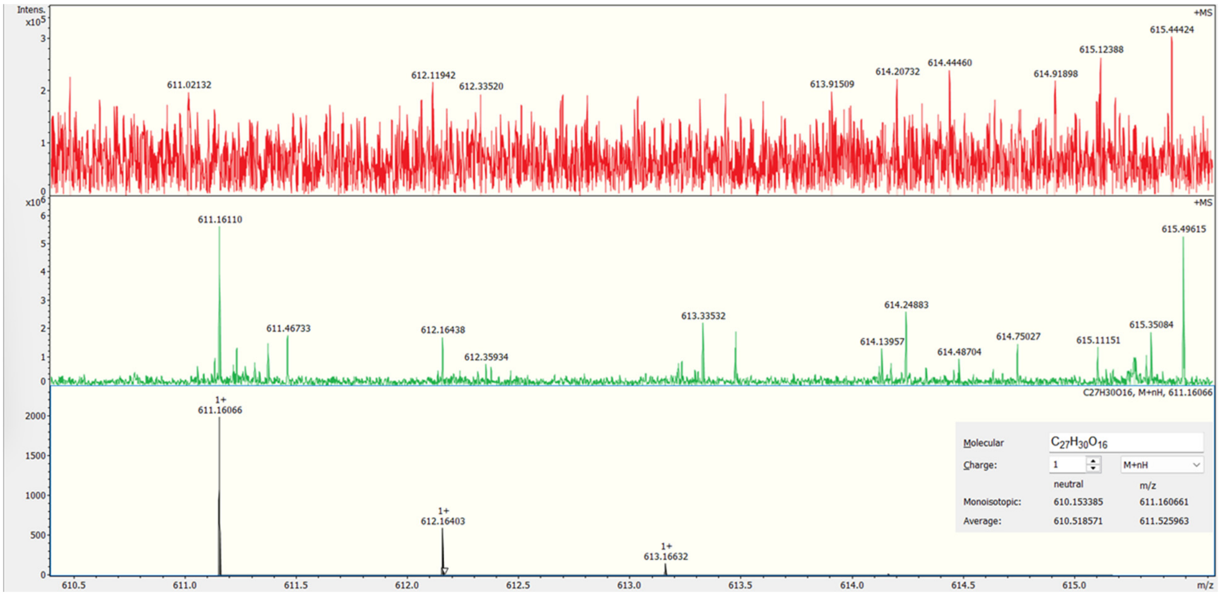

**Figure S15.** Molecular peak for Rutin ($C_{27}H_{30}O_{16}$) – m/z calculated is 611.160, ESI positive

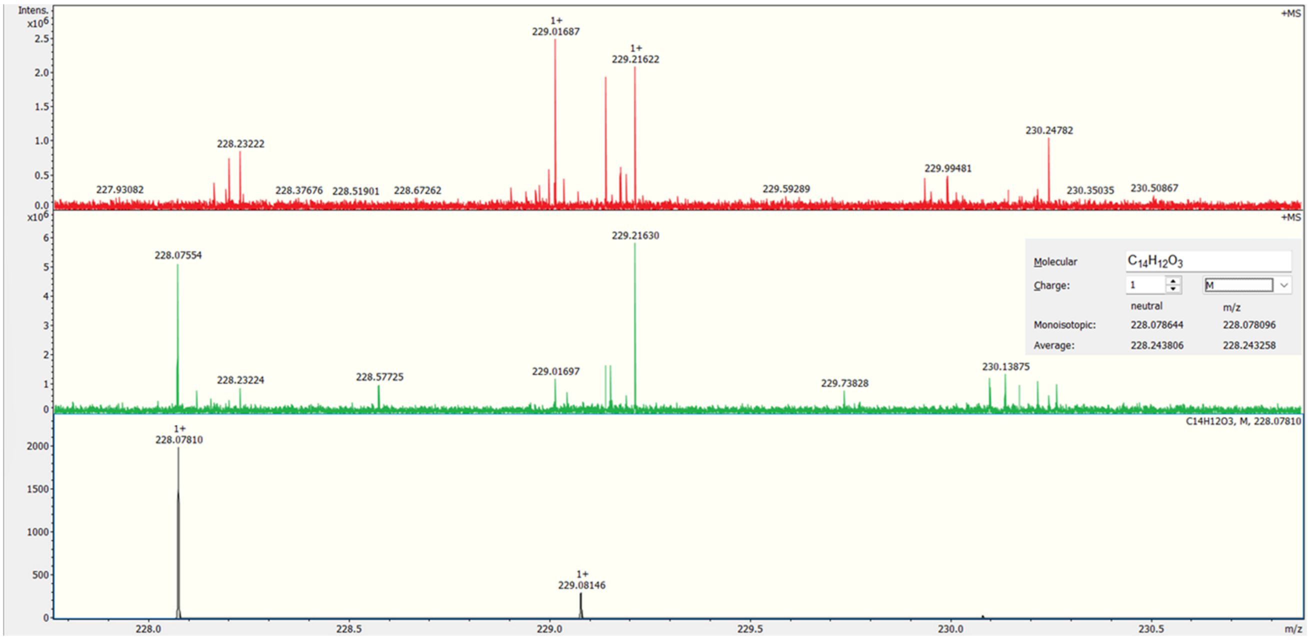

**Figure S16.** Molecular peak for t-Resveratrol ($C_{14}H_{12}O_3$) – m/z calculated is 228.078, ESI positive
